# Quality of Life in POP: Validity, Reliability and Responsiveness of the Prolapse Quality of Life Questionnaire (P-QoL) in Spanish Women

**DOI:** 10.3390/ijerph17051690

**Published:** 2020-03-05

**Authors:** Beatriz Sánchez-Sánchez, Maria José Yuste-Sánchez, Beatriz Arranz-Martín, Beatriz Navarro-Brazález, Helena Romay-Barrero, María Torres-Lacomba

**Affiliations:** 1 Physiotherapy in Women’s Health (FPSM) Research Group, Physiotherapy Department, Faculty of Medicine and Health Sciences, University of Alcalá, Alcalá de Henares, 28805 Madrid, Spain; beatriz.sanchez@uah.es (B.S.-S.); marijo.yuste@uah.es (M.J.Y.-S.); beatriz.arranz@edu.uah.es (B.A.-M.); maria.torres@uah.es (M.T.-L.); 2 Faculty of Physiotherapy and Nursing, University of Castilla-La Mancha, 45071 Toledo, Spain

**Keywords:** Spanish validation, pelvic organ prolapse, quality of life, P-QoL, validity, reliability, responsiveness

## Abstract

The Prolapse Quality of Life Questionnaire (P-QoL) is a specific questionnaire created to assess the impact of pelvic organ prolapse on women’s quality of life. The aim of the present study was to cross-culturally adapt and assess the psychometric properties of the P-QoL for Spanish women. The cross-cultural adaptation was conducted by a standardized translation/back-translation method. Psychometric analysis was performed by assessing the validity, reliability, responsiveness and feasibility. A total of 200 Spanish women were recruited and assigned to symptomatic and asymptomatic groups. The Spanish P-QoL version demonstrated good content validity. Convergent validity showed high intercorrelations with the Pelvic Floor Distress Inventory short form and the Pelvic Floor Impact Questionnaire short form. The discriminant validity showed statistically significant differences between the symptomatic and the asymptomatic groups. The internal consistency was high and of acceptable values. The test-retest reliability was shown to be high in all the cases. Regarding responsiveness, the effect size and standardized response mean demonstrated moderate values. The average time for administration was 10 (3) min. The Spanish P-QoL showed considerable support for the appropriate metric properties of validity, reliability, responsiveness and feasibility to evaluate the symptom severity and its impact on the quality of life in Spanish women with urogenital prolapse.

## 1. Introduction

Pelvic organ prolapse (POP) is defined as the symptomatic descent of one or more of the anterior vaginal walls, the posterior vaginal wall and the apex of the vaginal (uterus or vault) or uterus [1]. It is a common condition affecting approximately 30% of women between 20–59 years of age and more than half of women over 50 years of age attending gynecologic clinics [2]. Women with POP often complain of “feeling a bulge in the vagina” or “feeling pressure in the pelvis”. POP often coexists with other pelvic floor disorders, such as lower urinary tract, bowel and sexual dysfunction [3]. 

Although POP does not carry any risk to life, it is associated with significant morbidity. Women with POP have great symptomatic distress and impaired quality of life, causing physical, social, psychological, occupational, domestic and/or sexual limitations [2,4]. The severity and the impact of the prolapse symptoms on the quality of life are important for the clinical assessment to design more adequate treatment. A valid way to evaluate the patient´s condition and treatment effect is the condition-specific health-related quality of life questionnaires. Some specific tools were created to measure the subjective perception of quality of life and symptoms in women with pelvic floor disorders. The Pelvic Floor Distress Inventory short form (PFDI-20) and the Pelvic Floor Impact Questionnaire short form (PFIQ-7) are self-reported questionnaires that can address the presence and severity of pelvic floor disorders’ symptoms and their impact on quality of life [5]. Although these tools have been adapted and validated for Spanish women [6], they are generic instruments measuring the impact of pelvic floor disorders in general dysfunction (urinary, anal, bowel and sexual), they do not specifically assess quality of life in POP. So, there is a lack of validated quality of life tools for pelvic organ prolapse in women in Spain.

The Prolapse Quality of Life Questionnaire (P-QoL) is a simple, reliable and easily comprehensible instrument to assess the severity of POP symptoms and its impact on women’s quality of life. Moreover, it also provides treatment outcomes in women with POP, the applicability of which to clinical practice is widespread. It allows the clinicians to have the capacity to correlate symptoms with actual physical findings. The P-QoL was developed in 2005 by Digesu et al., and it was created for an English-speaking population [2]. It was cross-culturally adapted and validated in different countries (e.g., Italy, Turkey, Slovakia, Brazil, Germany, Taiwan, Iran, Chile) [7], so, it is only available in these countries. It contains 38 simple questions representing all important quality of life domains for the concept of urogenital prolapse and each domain is related to a particular aspect of quality of life: general health, prolapse impact, role, physical and social limitations, personal relationships, emotional problems, sleep/energy disturbance as well as severity symptoms’ measurements.

The aim of the present study has been to adapt and evaluate the psychometric properties of validity, reliability, responsiveness and feasibility of the Spanish version of the P-QoL questionnaire to considerate it as a reference instrument to assess symptom severity and quality of life in women with POP in Spain.

## 2. Materials and Methods 

### 2.1. Translation and Cultural Adaptation

This is a cross-cultural adaptation and psychometric validation study conducted between March 2013 and February 2017. This study was approved by Príncipe de Asturias University Hospital Clinical Research Ethics Committee in Alcalá de Henares (Madrid) (OE10/2010), Spain. 

The cross-cultural adaptation was developed in three phases, according to the International Society for Pharmacoeconomics and Outcomes Research (ISPOR) Task Force for Translation and Cultural Adaptation [8]. First, in order to obtain semantic, conceptual, idiomatic and content equivalence, the P-QoL original version in English was translated into Spanish by two English-Spanish translators, native Spanish-speaking, who worked independently to produce two Spanish versions that were conceptually equivalent to the original questionnaires. This translation was reviewed by these translators and the research team agreed with the Spanish translation synthesis. Afterwards, to obtain the back-translated version, two bilingual professional Spanish-English translators, native English-speaking, worked independently to produce two English versions of the Spanish questionnaire obtained in the forward translation. With these back-translated versions and with the translated version, an Expert Committee agreed the preliminary P-QoL Spanish version was equivalent to the original instrument. Afterwards, the P-QoL Spanish preliminary version was tested in a pilot study for comprehensibility, readability and equivalence through its administration to 20 native Spanish-speaking women who fulfilled the inclusion criteria. The women filled in the questionnaire on their own, and afterwards, they were interviewed face to face in order to identify and correct potential understanding difficulties of the items, as well as to judge the quality of the cultural adjustment. Minor discrepancies were identified and amended, and eventually, the P-QoL Spanish version was obtained.

### 2.2. Participants and Procedure

All women who were assessed in the Physiotherapy in Women’s Health Research Unit of the Alcalá University (Madrid, Spain)and that fulfilled the inclusion criteria were informed about the study and invited to participate. Thus, written informed consent was obtained from all the participants. The inclusion criteria were: aged above 18 and being able to read and to understand Spanish. The exclusion criteria were: current pregnancy, neurological diseases or mental incapacity to properly fill in the questionnaire. 

At baseline, women who agreed to participate in the study completed the Spanish versions of the following instruments: P-QoL, PFDI-20, PFIQ-7 and a sociodemographic and clinic form. These questionnaires were self-reported, and each of them had instructions on how to complete it. Subsequently, women were examined in supine position using the Pelvic Organ Prolapse Quantitation System (POP-Q), approved by the International Continence Society (ICS) [9]. According to POP symptoms of vaginal bulge or the feeling of something coming down or out of the vagina, women were categorized into two groups: symptomatic and asymptomatic.

Sample size was based on the recommendations of previous works [10,11], there being at least 100 subjects with a minimum of 50 participants for every subgroup analysis.

In order to analyze the test-retest reliability, a subsample of 31 symptomatic women, recruited consecutively, filled in the P-QoL again two weeks later. No treatment was delivered during this time; this interval was chosen to ensure that the women’s symptoms remained unchanged and for long enough to ensure that they could not recall their baseline responses. In order to analyze the responsiveness, 39 symptomatic women recruited consecutively, were assessed after completing a two-month physiotherapy program. The average time was recorded.

### 2.3. Instruments

The P-QoL questionnaire is a self-reported tool that contains 20 questions representing nine quality of life domains: general health (1 item), prolapse impact (1 item), role limitations (2 items), physical limitations (2 items), social limitations (3 items), personal relationships (2 items), emotions (3 items), sleep/energy (2 items) and severity measures (4 items). Scores in each domain range from 0 to 100. Higher scores indicate a greater impairment of quality of life, and lower scores indicate a good quality of life. Additionally, there are 18 questions regarding urinary, bowel, and prolapse/vaginal symptoms, which don not have an assigned score. Response options are on a Likert scale with ranges between 1 and 4, except for the first question, that ranges between 1 and 5. Symptoms questions and personal relationships domains offer the option *Not applicable*. The original version of the P-QoL reported reliability (Cronbach´s alpha greater than 0.80 and high test-retest reliability) and discriminant validity (statistically significant differences between symptomatic and asymptomatic group). 

The PFDI-20 and the PFIQ-7 are recommended tools to assess the presence of symptoms and the impact on health-related quality of life in the three domains: POP, bowel and urinary [12,13]. The PFDI-20 contains 20 questions divided in three domains: genital prolapse symptoms, colorectal-anal symptoms and urinary symptoms. The PFIQ-7 consists of seven questions covering the effect on activities, relationships or feelings of each symptom: urinary, colorectal-anal and genital prolapse. In both instruments, the minimum score for each block is 0 points (low involvement), and the maximum, 100 points (maximum effect). The PFDI-20 and the PFIQ-7 are highly recommended (grade A) for the evaluation of symptoms and health-related quality of life impact of POP by the International Consultation on Incontinence (ICI) [14].

Women’s characteristics recorded were: age, weight, height, educational level, parity, vaginal delivery and physical examination (classification of POP-Q scale).

### 2.4. Psychometric Validation. Statistical Analysis

The P-QoL Spanish version was assessed for validity, reliability, responsiveness and feasibility.

Analysis was conducted using SPSS version 24.0. Statistical significance was assumed at *p* < 0.005. Consensus-based Standards for the selection of health Measurement Instruments (COSMIN) recommendations were used as a guide for evaluating the psychometric properties [15]. 

The sociodemographic and clinical data between the symptomatic and asymptomatic women were compared. Student’s *t*-test was used to compare age and body mass index, the chi-square test was used for vaginal delivery and education level, and Fisher’s exact test was used for level of parity and Pelvic Organ Prolapse Quantitation stages.

Validity was assessed by the content and construct validity (convergent and discriminant). Although content validity for assessing the ability of items to collect health status was guaranteed by the validation of the original scale, in this study the Expert Committee’s opinion was also considered to judge the ability of the questionnaire to cover all items for the concept of urogenital prolapse symptoms and quality of life, and the feedback of the pilot study of 20 women, who answered the questionnaires and were asked to indicate if they had any difficulty or found any ambiguous words or phrases. 

The construct validity is the extent to which scores demonstrate expected logical relations with other variables. It was measured with the correlations between the P-QoL domains scores, the PFDI-20, and the PFIQ-7 domains. Total scores in the symptomatic group were evaluated using the Spearman correlation (r). Validity was considered high when the range was included between 0.30 and 0.40 (10). Expecting the highest correlations between P-QoL and POP dimensions of the PFDI-20 and PFIQ-7 (POPDI and POPIQ, respectively).

With regard to the discriminant construct validity, we assessed whether the Spanish version was able to discriminate between symptomatic and asymptomatic women. For this purpose, an independent Student´s *t*-test was calculated, comparing the difference of the scores of P-QoL domains between both groups using the Mann–Whitney *U* test.

Reliability was assessed by the internal consistency and the test-retest reliability. Internal consistency (the degree of interrelatedness among the items [15]) was measured by means of Cronbach’s alpha, considering a value of 0.70 a good reliability; the higher the value, the greater the internal consistency [8,11]. The test-retest reliability (the degree to which a measurement is free of error [15]) was assessed by the intraclass correlation coefficient (ICC). Good values of test-retest reliability are considered greater than 0.70 [11]. A test-retest analysis of two weeks was used. 

The responsiveness or sensitivity to change was assessed in a subgroup of symptomatic women that received a physiotherapy treatment for the pelvic floor for 8 weeks. These women filled in the P-QoL twice: at baseline, and after the physiotherapy intervention. In order to evaluate responsiveness, three distribution-based methods were used: the *p* values generated using the Wilcoxon signed-rank test, the effect size (ES), and the standardized response means (SRM) for the change in scores from pre- and post-physiotherapy intervention using the paired t test. For SRM and ES, a value of 0.2–0.5 was considered as small, one of 0.5–0.8 as moderate, one of above 0.8–1.0 as good, and one of more than 1.0, excellent [16,17,18].

To evaluate the P-QoL Spanish version’s feasibility, the percentage of unanswered individual items and the percentage of patients who did not answer any of the items were analyzed. Also, the average administration time was calculated.

## 3. Results

### 3.1. Study Participants’ Characteristics

We enrolled 222 women; of these, 22 (9.9%) did not meet the inclusion criteria or declined to participate. In the end, 200 women completed the study. A hundred (100%) women were symptomatic and 100 (100%) were asymptomatic. Sociodemographic characteristics and POP stage based on Pelvic Organ Prolapse Quantitation examination for symptomatic and asymptomatic groups are shown in Table 1. 

All the sociodemographic and clinical characteristics were significant except for the body mass index (BMI, Table 1). The median age was 52 (13.4) and 40.6 (11.1) for symptomatic and asymptomatic groups, respectively (*p* < 0.001). With regard to the Pelvic Organ Prolapse Quantitation stage, the majority of women were stage II in the symptomatic group, 76 (76%), while those in the asymptomatic group, 72 (72%), were stage 0 (*p* < 0.001).

### 3.2. Cross-Cultural Adaptation 

The revisions of experts and the women in the pilot study guaranteed the content validity in the P-QoL Spanish version. Most of the items were well understood by the women. No words or items showed difficulty in their comprehensibility and did not need to be adapted to Spanish culture, almost all women found the questionnaire easy to understand and relevant. The final version of the Spanish P-QoL maintains the structure of the original version, included the 20 questions representing nine quality of life domains (general health, prolapse impact, role limitations, physical limitations, social limitations, personal relationships, emotions, sleep/energy and severity measures), and the 18 questions about the bladder, bowel and sexual function (Table S1 and Table S2 Supplementary Material).

All the questionnaires were filled in by the women in the clinic. Therefore, there were no missing data and the majority of items were easily understood. 

### 3.3. Validity 

To guarantee an adequate content validity, the Spanish version of P-QoL was reviewed by the Expert Committee and the pilot study. The Expert Committee agreed that the questionnaire included all the relevant dimensions to assess the impact of POP, and the interviewed women indicated that the items were well understood, and that the questionnaire showed a good readability and comprehensibility.

Regarding the convergent construct validity, all P-QoL dimensions showed high values with the total PFDI-20 and PFIQ-7, the lowest values corresponding to “personal relations”. The correlations were higher in the total PFIQ-7 than in the total PFDI-20. With regard to the correlations within the domains, as expected, the POP domains (POPDI and POPIQ) that were highest, and those that were lowest, were the bowel domains (Table 2).

Discriminative construct validity showed that the total scores for each P-QoL domains (median (IQR)) differed significantly between the symptomatic and the asymptomatic women (*p* < 0.001 and *p* = 0.003), with the lowest differences in the “General Health Perceptions” (Table 3).

### 3.4. Reliability

In terms of reliability, internal consistency and test-retest were calculated (Table 4). High internal consistency showed in all dimensions with a Cronbach’s alpha coefficient range between 0.751 to 0.877, except for the “sleep/energy” and “severity measures” dimensions, which were shown to be acceptable (0.621 and 0.550, respectively). The score of the internal consistency in “general health perceptions” and “prolapse impact” domains could not be calculated, because both domains only have one item. For the test-retest reliability, 35 women from the symptomatic group were invited to complete the questionnaire two weeks after the baseline assessment, and 31 women completed this test-retest correctly. The test-retest reliability was high in all cases, with values from 0.725 to 0.938. All the values were statistically significant (*p* < 0.001). 

### 3.5. Responsiveness

To assess responsiveness, a total of 39 women were recruited from the symptomatic group. With regard to the baseline characteristics of these women, the median age was 43 (9) and the 34 (87.2%) were Pelvic Organ Prolapse Quantitation grade II. The ES and SRM were moderate in all domains except in “General Health Perceptions”, which were small. The highest value was that of the “Prolapse Impact” domain (ES 0.71 and SRM 0.73; *p* < 0.001) (Table 5).

### 3.6. Feasibility

Concerning feasibility, the average time for questionnaire administration was 10 (3) min for the P-QoL Spanish version. The women with non-response items were zero in the nine dimensions. 

## 4. Discussion

Clinical management of POP should consider the severity of symptoms and the impact on women’s quality of life to select the most appropriate management in each case, surgical or conservative treatment; as well as to evaluate its effectiveness [2]. In fact, improvement in quality of life should be the main aim of any prolapse treatment. The assessment of this impact by clinicians may be difficult or inaccurate due to the women’s embarrassment during clinical assessment. There are three Spanish validated questionnaires that assess the impact of pelvic floor disorders in women: the PFDI-20, the PFIQ-7, and the Pelvic Organ Prolapse Incontinence Sexual Questionnaire, International Urogynecological Association (IUGA)-revised. Although they are generic tools to measure the impact of pelvic floor disorders in general dysfunction (urinary, anal, bowel and sexual), they do not specifically assess quality of life in POP. The P-QoL is a specific “self-reported” questionnaire to assess the impact of POP on women’s quality of life that covers different domains of women lifestyle. 

The P-QoL questionnaire is easily administered to be used in clinical and research practice. It was developed and validated for the English language and has been adapted to and validated in numerous languages [7]. Our goal has been to validate a tool to evaluate the prolapse-related symptoms and its impact on the quality of life of Spanish women. 

The translation/back translation method used for this Spanish version is similar to that performed in the other validity versions of P-QoL. The linguistic adaptation process showed that women easily understand the P-QoL Spanish version, and did not report any difficulty, which could be due to the questions and their answers being simple. Our version did not need to be adapted to Spanish culture, finding relevant all the particular aspect of quality of life included. In fact, the original P-QoL was created for a European population. The Spanish version maintains the meaning, intent and structure of the original English version.

To our knowledge, the present Spanish P-QoL is the only specific instrument that has been adapted and validated to assess the impact on quality of life of pelvic organ prolapse in Spain. In spite of there being a validated Spanish version of P-QoL in Chilean women, it could not be used in Spanish women from Spain, because even if it is the same language, there are different expressions, contexts and cultures, so, the linguistic adaptation does not consist of literal translation, but rather of developing conceptually equivalent and cultural appropriate versions adapted to the target country. So, the validation of an instrument is necessary in order to be used in different countries with the same language to check the linguistic validation [19,20]. Secondly, it is essential to assess the psychometric properties of the validated tool to the target population, in our case, Spanish women from Spain. Therefore, using a tool validated for other country in a Spanish population may be a significant source of bias in the data obtained. 

In this way, the validation of P-QoL in Spain was necessary in order to have a tool that assesses the severity of the symptoms and their impact on quality of life in women with POP, to guide professionals and researchers in the choice of instruments with universal application. In fact, the use of the same tool for data collection allows the possibility of international multicentre studies and comparing results of studies from different countries, allowing meta-analysis of the published results [14].

Sociodemographic characteristics showed that the sample was similar to other validations of this questionnaire, and the average age was higher in the symptomatic than in the asymptomatic group. Although there are differences between the two groups, this data does not interfere in the final conclusions. Regarding the stage prolapse, in our study we found that 95% of the symptomatic women had POP stages II to IV, while 82% of the asymptomatic women had stages 0 to I. This can be justified because most often women become symptomatic when the prolapse is stage III or IV, and sometimes in II [21]. The results of our study confirm that most symptomatic women had stage II or above, while asymptomatic women had no more than II. 

Regarding the convergent construct validity, the comparison of P-QoL with the PFDI-20 and PFIQ-7 was evaluated. Both instruments are highly recommended (grade A) by ICI for the evaluation of symptoms and the health-related quality of life impact of POP [14], making them an appropriate comparison tool for the P-QoL; in fact, the Chinese version of P-QoL has recognized the absence of these criteria in their study to be a limitation. In the symptomatic group, we found a correlation between the P-QoL and the total the Pelvic Floor Distress Inventory short form and the Pelvic Floor Impact Questionnaire short form, and the highest correlations with the POP dimensions, followed by urinary dimensions. This may reflect the fact that the lower urinary tract symptoms can be usually triggered by the compression from the kinking of a POP. 

Some dimensions of our questionnaire showed low scores, which could be due to the fact that most of the women included in the symptomatic group were ≤grade II of prolapse (81%) and some studies report worse quality of life with increasing severity of pelvic organ prolapse [22,23,24]. Despite this, our questionnaire was able to discriminate between symptomatic and asymptomatic women. We found the total scores for each domain of P-QoL to be significantly higher for symptomatic women compared to asymptomatic women (*p* value < 0.05). The “general health perceptions” showed the lowest difference, which is in line with the P-QoL original and other versions like the Dutch and Portuguese [25,26]. In the asymptomatic, all domains except general health and sleep/energy domains had a score of zero, which was justified due to all domains evaluating the impact of quality of life specifically due to POP, except “general health” and “sleep/energy”. The “general health” domain contains a unique question that asks about health in general, and general health perception can be affected by other pathological conditions or diseases not related to POP, so it is not a specific prolapse condition. In fact, the general health of a woman with no prolapse but with any other medical conditions might be affected, while a woman with prolapse might have good general health. Regarding the “sleep/energy” dimension, the second item asks if she feels tired, and again, this is not a specific condition related to POP, since a woman could be tired for multiple reasons. The main difference that we found between the two groups was in the “prolapse impact” domains, which is logical, because the only question that evaluates it asks directly about how much the prolapse affects the woman’s life, so this is a specific item of prolapse condition.

The P-QoL Spanish version showed high internal consistence in all dimensions except in the “sleep/energy” and “severity measures” dimensions, which were the lowest, as has been reported in other validations like Portuguese (Brazilian), Persian or Slovakian [26,27]. Cronbach´s alpha will generally increase as the intercorrelations among items increase, when all items measure the same construct. A low Cronbach´s alpha suggests that these dimensions are testing different traits [28]. Regarding test-retest reliability, it was high in all the dimensions.

It is widely argued that outcome measures in clinical trials should show not only validity and reliability, but also responsiveness [29]. Responsiveness or sensitivity to change is the ability to detect changes that occur as a result of therapy or disease progression and has been suggested as one criterion to choose among scales used to evaluate the efficacy of a therapeutic intervention. In fact, a low response can induce a lack of difference when a real difference exists (type II error), which can lead to an underestimation on the effect of treatment [30]. As we know, the Dutch (Belgium), Persian (Iran), Portuguese (Brazil) and the present study, are the only P-QoL validations that have evaluated responsiveness [25,28,31]. All these validations assessed the responsiveness by comparing pre- and post-operative scores, but the Spanish (Spain) version evaluated it with pre- and post-physiotherapy, which implies that we can detect the responsiveness of P-QoL to assess changing quality of life with a small change in score after physiotherapy. Responsiveness is of particular importance in urogynecology, where the primary aim of an intervention is mostly to improve the individual’s quality of life [32]. Therefore, we consider responsiveness to be a fundamental psychometric characteristic that should be assessed in our study. The P-QoL Spanish responsiveness showed the highest scores in the prolapse impact domain and the lowest scores in the general health domain, which is in accordance with the fact that this domain only contains one question about general health which is not specifically related to the prolapse condition, so the treatment of POP may not improve it. 

There were some limitations to this study. Most of the women included in the symptomatic group had a low grade of prolapse (81% were ≤grade II). Some studies report worse quality of life with increasing severity of pelvic organ prolapse. Therefore, it would be recommendable to support the psychometric properties in women with a high grade of prolapse [22,23,24]. In the present study, responsiveness was assessed in women undergoing a physiotherapy treatment, which implies that the changes can be lower than those seen after surgical treatment, so, we found moderate responsiveness and further studies are needed to evaluate responsiveness comparing pre- and postoperative scores. 

## 5. Conclusions

In conclusion, these results show considerable support to the appropriate metric properties of reliability, validity, responsiveness and feasibility of the P-QoL Spanish version to provide a useful instrument to evaluate symptom severity and their impact on quality of life related to prolapse in Spanish women. It is a well understood and easily administered instrument that should be recommended for routine use in clinical practice to better identify those women who need treatment for POP and to assess outcomes by a comparison between pre- and post- treatment.

## Figures and Tables

**Table 1:** Sociodemographic and clinical characteristics.

	Symptomatic (N = 100)	Asymptomatic (N = 100)	*P* Value
Age (years, X (SD))	52 (13.4)	40.6 (11.1)	<0.001 *
Parity (n (%))			<0.001 ***
0	4 (4.1)	4 (4.2)	
1	16 (16.6)	56 (58.3)	
2	46 (47.4)	26 (27.1)	
≥3	31 (32)	10 (10.4)	
Vaginal delivery (n (%))			<0.001 **
0	7 (7.2)	6 (6.3)	
1	20 (20.6)	57 (59.4)	
2	48 (49.5)	27 (28.1)	
≥3	22 (22.7)	6 (6.3)	
Body mass index (BMI)	26.7 (4.4)	24.7 (4.2)	0.028 *
(kg/m^2^, X (SD))			
Education level (n (%))			<0.001 **
Primary School	44 (44.4)	17 (17)	
High School	28 (28.3)	27 (27)	
College or University	27 (27.3)	56 (56)	
POP-Q stage (n (%))			<0.001 ***
Stage 0	0 (0)	72 (72)	
Stage 1	5 (5)	10 (10)	
Stage 2	76 (76)	18 (18)	
Stage 3	12 (12)	0 (0)	
Stage 4	7 (7)	0 (0)	

* Student *t*-test; ** Pearson chi-square test, *** Fisher’s exact test. X (SD): Mean (Standard Deviation); POP-Q: Pelvic Organ Prolapse Quantitation.

**Table 2:** Results from the analysis of convergent construct validity (Spearman´s coefficient (r)) for Prolapse Quality of Life Questionnaire (P-QoL) with Pelvic Floor Impact Questionnaire (PFIQ-7) and Pelvic Floor Distress Inventory (PFDI-20) total and dimensions (N = 100).

Prolapse Quality of Life Domain	UIQ	CRAIQ	POPIQ	TOTAL PFIQ-7	POPDI	CRADI	UDI	TOTAL PFDI-20
General Health P.	0.398(**)	0.356(**)	0.320(**)	0.440(**)	0.345(**)	0.396(**)	0.361(**)	0.468(**)
Prolapse Impact	0.489(**)	0.274(**)	0.537(**)	0.547(**)	0.414(**)	0.131	0.513(**)	0.468(**)
Role Limitations	0.412(**)	0.313(**)	0.532(**)	0.537(**)	0.442(**)	0.172	0.405(**)	0.410(**)
Physical Limitations	0.505(**)	0.345(**)	0.555(**)	0.617(**)	0.412(**)	0.180	0.442(**)	0.391(**)
Social Limitations	0.488(**)	0.411(**)	0.517(**)	0.543(**)	0.390(**)	0.330(**)	0.474(**)	0.453(**)
Personal Relationships	0.343(**)	0.204	0.306(**)	0.364(**)	0.266(*)	0.215	0.235(*)	0.265(*)
Emotions	0.436(**)	0.253(*)	0.511(**)	0.567(**)	0.380(**)	0.216(*)	0.308(**)	0.357(**)
Sleep/Energy	0.454(**)	0.399(**)	0.305(**)	0.432(**)	0.324(**)	0.326(**)	0.324(**)	0.351(**)
Severity Measures	0.316(**)	0.252(*)	0.392(**)	0.441(**)	0.444(**)	0.187	0.440(**)	0.462(**)

*p < 0.05 is considered statistically significant. **p < 0.01 is considered statistically significant. UIQ: Urinary Impact Questionnaire; CRAIQ: Colorectal-Anal Impact Questionnaire; POPIQ: Pelvic Organ Prolapse Impact Questionnaire; PFIQ-7: Pelvic Floor Impact Questionnaire short form; POPDI: Pelvic Organ Prolapse Distress Inventory; CRADI: Colorectal-Anal Distress Inventory; UDI: Urinary Distress Inventory; PFDI-20: Pelvic Floor Distress inventory short form.

**Table 3:** Discriminative validity. Correlation of P-QoL domains scores between symptomatic and asymptomatic women by Mann–Whitney test.

Prolapse Quality of Life Domain	Symptomatic (N = 100)	Asymptomatic (N = 100)	
	Md (IQR)	Md (IQR)	*P* Value
General Health Perceptions	25 (25)	25 (0)	0.003
Prolapse Impact	33.3 (33.3)	0 (0)	<0.001
Role Limitations	0 (33.33)	0 (0)	<0.001
Physical Limitations	0 (33.33)	0 (0)	<0.001
Social Limitations	0 (11.1)	0 (0)	<0.001
Personal Relationships	0 (33.3)	0 (0)	<0.001
Emotions	0 (33.3)	0 (0)	<0.001
Sleep/Energy	16.7 (33.3)	0 (16.7)	<0.001
Severity Measures	16.7 (25)	0 (0)	<0.001

*p* < 0.05 is considered statistically significant. Md (IQR): Median (interquartile range).

**Table 4:** Reliability of P-QoL domain scores.

Prolapse Quality of Life Domain	Test-Retest Reliability (N = 31)		Internal Consistency
	ICC	*P* value	Cronbach´s Alpha
General Health Perceptions	0.791	<0.001	-
Prolapse Impact	0.862	<0.001	-
Role Limitations	0.863	<0.001	0.848
Physical Limitations	0.908	<0.001	0.768
Social Limitations	0.864	<0.001	0.751
Personal Relationships	0.725	<0.001	0.806
Emotions	0.849	<0.001	0.877
Sleep/Energy	0.938	<0.001	0.621
Severity Measures	0.857	<0.001	0.550

ICC: interclass correlation coefficients.

**Table 5:** P-QoL mean change in scores and responsiveness (N = 39).

Prolapse Quality of Life Domain	Pretreatment *X* (SD) Score	Posttreatment *X* (SD) Score	*P* Value	Mean Change in Score (SD)	Effect Size (ES)	Standardized Response Mean (SRM)
General Health P.	30.77 (20.24)	25 (16.22)	0.083	(20.24) 5.77	0.29	0.29
Prolapse Impact	40.17 (28.79)	19.65 (15.03)	<0.001	(28.21) 20.52	0.71	0.73
Role Limitations	21.36 (27.29)	9.4 (14.70)	0.004	(24.16) 11.96	0.44	0.50
Physical Limitations	29.05 (30.52)	11.96 (17.07)	<0.001	(27.7) 17.09	0.56	0.62
Social Limitations	16.52 (24.29)	3.13 (8.04)	<0.001	(21.95) 13.39	0.55	0.61
Personal Relationships	26.92 (31.44)	8.97 (17.45)	<0.001	(31.38) 17.95	0.57	0.57
Emotions	28.49 (29.15)	9.97 (17.25)	<0.001	(29.43) 18.52	0.64	0.63
Sleep/Energy	25.64 (26.44)	12.39 (18.62)	<0.001	(20.29) 13.25	0.50	0.65
Severity Measures	20.72 (19.2)	8.33 (13.24)	<0.001	(16.87) 12.39	0.65	0.73

*p* < 0.05 is considered statistically significant. *X* (SD): Mean (Standard Deviation); SD: standard deviation; ES effect size; SRM: standardized response means.

## Supplementary Materials

The following are available online at https://www.mdpi.com/1660-4601/17/5/1690/s1: Table S1: Spanish P-QoL; Table S2: Spanish P-QoL scoring.
